# Convergence of Scaffold-Guided Bone Reconstruction and Surgical Vascularization Strategies—A Quest for *Regenerative Matching* Axial Vascularization

**DOI:** 10.3389/fbioe.2019.00448

**Published:** 2020-01-10

**Authors:** David S. Sparks, Flavia Medeiros Savi, Siamak Saifzadeh, Michael A. Schuetz, Michael Wagels, Dietmar W. Hutmacher

**Affiliations:** ^1^Centre for Regenerative Medicine, Institute of Health and Biomedical Innovation, Queensland University of Technology, Kelvin Grove, QLD, Australia; ^2^Department of Plastic & Reconstructive Surgery, Princess Alexandra Hospital, Woolloongabba, QLD, Australia; ^3^Southside Clinical Division, School of Medicine, University of Queensland, Woolloongabba, QLD, Australia; ^4^Medical Engineering Research Facility, Queensland University of Technology, Chermside, QLD, Australia; ^5^Department of Orthopaedic Surgery, Royal Brisbane Hospital, Herston, QLD, Australia; ^6^Jamieson Trauma Institute, Royal Brisbane Hospital, Herston, QLD, Australia; ^7^Australian Centre for Complex Integrated Surgical Solutions, Woolloongabba, QLD, Australia; ^8^ARC Centre for Additive Bio-Manufacturing, Queensland University of Technology, Kelvin Grove, QLD, Australia

**Keywords:** tissue engineering, vascularization, bone, regeneration, blood vessel analysis

## Abstract

The prevalent challenge facing tissue engineering today is the lack of adequate vascularization to support the growth, function, and viability of tissue engineered constructs (TECs) that require blood vessel supply. The research and clinical community rely on the increasing knowledge of angiogenic and vasculogenic processes to stimulate a clinically-relevant vascular network formation within TECs. The regenerative matching axial vascularization approach presented in this manuscript incorporates the advantages of flap-based techniques for neo-vascularization yet also harnesses the *in vivo* bioreactor principle in a more directed “like for like” approach to further assist regeneration of the specific tissue type that is lost, such as a corticoperiosteal flap in critical sized bone defect reconstruction.

## Introduction

The management of critical size bone defects is still a challenge to the reconstructive surgeon in the twenty first century (Wagels et al., 2013). These defects may arise after trauma or oncological bone resection. The goal of limb salvage is restoration of bone stock to facilitate mechanical loading within a reasonable time period. Historically, this can be achieved by limb shortening, non-vascularized autograft, the Masquelet technique (autograft placed into an induced membrane), distraction osteogenesis and vascularized bone with or without allograft. Unfortunately evidence-based medicine has not generated the datasets yet to establish a clinical guide as to when any given technique is preferred over another. The value of limb salvage to both the patient and the community in which they live seems self-evident, yet it has become increasingly clear that successful limb salvage can outperform amputation and prosthetic rehabilitation after prolonged follow up (Bosse et al., 2002; Chung et al., 2009; Higgins et al., 2010).

With the increasing clinical relevance of translating tissue engineering into regenerative medicine (Warnke et al., 2004), more tailored options for tissue reconstruction are emerging. Twenty first centuries biomaterial-based methods, including well-designed and fabricated biodegradable scaffolds, are at the forefront of this frontier (Reichert et al., 2012) and may facilitate reconstruction of lost tissues with high fidelity in the future. Recent clinical experience in the reconstruction of a variety of critical size bone defects across a range of anatomical regions including the mandible (Warnke et al., 2004; Kokemueller et al., 2010; Wiltfang et al., 2016), maxilla (Mesimäki et al., 2009), radius (Horch et al., 2014), and tibia (Horch et al., 2014) has been encouraging. However, caution should be exercised in proposing from small animal data sets incorporating novel technology and techniques into mainstream surgical practice. The behavior of scaffold-based reconstruction for critical bone defects is plagued by the inherent difficulty in attaining uniform neo-vascularization, and therefore, uniform bone regeneration. This is perhaps an overlooked limitation to current techniques that are used in isolation without a focus on creating a vascular axis. Scaffold designs which do not allow physiological vascularity, particularly when living cellular material has been used, are prone to failure (Horch et al., 2012). In order to understand why this occurs, it is necessary to examine the relationship between vascularity and osteogenesis.

In the adult, new vascular growth into an auto- and allograft or scaffold from an existing host vascular bed occurs via angiogenesis (Carmeliet, 2003). This would appear to occur most predominantly via *luminal sprouting* from arterioles and venules that are in close proximity to the scaffold (Tanaka et al., 2003). In essence, a dominance of promotional factors over inhibitory factors leads to detachment of pericytes from endothelial cells, with the latter becoming activated by vascular endothelial growth factor (VEGF), amongst others (Beck and D'Amore, 1997). Endothelial cells then produce specific proteases that assist with the digestion of the vascular basement membrane and adjacent extra-cellular matrix (Risau, 1997). The endothelial cells then migrate externally from the vessel and proliferate to generate new capillaries that extend and sprout through the adjacent tissue/scaffold into arterioles/venules, driven by pro-angiogenic factors (Chen and Kaji, 2017). Through the myriad of hemorrhage, coagulation and inflammation associated with surgical manipulation, the process of angiogenesis can also be greatly enhanced (Risau, 1997).

Angiogenesis is intimately linked to osteogenesis (Ramasamy et al., 2014). Skeletal bone regeneration is underpinned by the sufficient delivery of osteoclast precursors and osteoprogenitor cells to the site of osseoinduction by invading blood vessels (Maes et al., 2010). Importantly, this process also involves cross-communication between long bone specific endothelial cells, chondrocytes and osteogeoprogenitor cells (Kusumbe et al., 2014). Agonistic communication derives from chondrocytes and osteoprogenitor cells involved in bone generation, which serve as a key source of VEGF-A (a pro-angiogenic mediator) (Ramasamy et al., 2014). Endothelial cells are stimulated by VEGF-A and up-regulate *Notch* activity, which is required for the expression of *Noggin*, a mediator that controls the differentiation of perivascular osteoprogenitor cells and with this osteogenesis (Ramasamy et al., 2014). Positive coupling between these mechanisms helps to facilitate both processes of osteogenesis and angiogenesis.

The following review applies the core patterns of tissue vascularization in reconstructive surgery to scaffold-guided bone regeneration. An emphasis is placed on the different methods for scaffold axial vascularization and a justification for the role of a *regenerative matching* approach with a corticoperiosteal flap to improve current cell-based bone regeneration strategies.

## Principles of Scaffold-Guided Bone Regeneration

Progress in bone engineering has been aided by the rise of additive manufacturing (3D printing), which allows the fabrication of scaffolds with customizable micro and macro-architecture in a number of different biodegradable materials. In scaffold-guided tissue regeneration (SGTR), cellular growth is mainly supported by the morphology and surface to volume ratio in a similar fashion to the role of the extra-cellular matrix in the physiological host environment (Hutmacher et al., 2004). Design principles for scaffold fabrication should be based on reproducible preclinical data sets of overall architecture and porosity, pore size, pore interconnections and surface to volume ratios. Specifically, porosity and pore size relate to the volume area available for host tissue in-growth, including vasculature, to penetrate into the central regions of the scaffold architecture. Increases in porosity alongside pore size and spacing of pore interconnectivity has been shown to positively influence tissue regeneration and this also correlates with scaffold surface area (Cipitria et al., 2012). In large preclinical animal models such as sheep, which closely simulate the human anatomical and physiological setting, pore interconnections smaller than 400 μm were found to restrict vascular penetration (Cipitria et al., 2012). In this way, a macroscopic channel—like pore architecture further stimulates physical tissue penetration. The ability of new blood vessels to grow into the tissue engineered construct (TEC) is also related to the pore size and morphology, thereby directly influencing the rate of in-growth of newly formed tissue into the TEC. As a general rule *in vivo*, larger pore sizes and interconnections (human applications > 600 microns) and higher porosity (>70%) lead to a faster rate of neo-vascularization, inherently leading to enhanced bone regeneration (Berner et al., 2015a; Cipitria et al., 2015). Yet, the animal model and the corresponding size and volume of the bone must be implemented in the design strategy. For example, the morphology and architecture of a scaffold for the regeneration of a mouse segmental femur defect deviates significantly from that which is seen in a sheep.

Another important consideration is the selection of scaffold material for bone regeneration, and is generally governed by the mechanical properties, degradation kinetics and biological interactions (protein adsorption, cellular attachment and consequently osteoconductivity) (Hutmacher et al., 2007). Composite scaffolds composed of synthetic analogs of the two major macro-molecular components that constitute normal bone (hydroxyapatite and collagen), such as polycaprolactone (PCL) with tricalcium phosphate (TCP) are commonly used due to their slow-degradability characteristics whilst maintaining strength and promoting osseoconduction over the entire remodeling period (Reichert et al., 2012; Berner et al., 2013, 2015a; Cipitria et al., 2013). Alternative scaffold materials include naturally occurring polymers (e.g., gelatin), synthetic bioresorbable polymers [e.g., poly(lactic-co-glycolic) acid, PGLA], synthetic porous ceramics (e.g., bioglass) and naturally occurring ceramics (e.g., coral). Generally speaking, if the desired end regenerate is bone, osseoconductive scaffold designs (PCL-TCP, collagen type-1 sponge with demineralized bone powder and calcium etc.) are a common choice in the literature for obvious reasons.

Further enhancement in the generation of new bone formation can also be attained through the addition of biological additives like growth factors (e.g., bone morphogenic protein-7, BMP-7) (Berner et al., 2012; Reichert et al., 2012; Cipitria et al., 2013, 2015) or cellular approaches (e.g., osteoprogenitor cell impregnation) (Reichert et al., 2012; Berner et al., 2015a). However, the success of growth factors and cellular impregnation techniques for scaffold-guided bone regeneration is intimately linked to vascularity within the construct and becomes particularly relevant for large segment bone volume loss. Without question, numerous factors must be considered when designing and fabricating scaffolds for applications in bone engineering. It is beyond the scope of this review to present all of them in detail and a number of comprehensive reviews have been published on this topic which we direct the reader to (Holzapfel et al., 2017; Bartnikowski et al., 2019).

## Patterns of Blood Supply in Scaffold-Guided Tissue Engineering

Introducing an arteriovenous pedicle to a scaffold effectively permits what is known as *axial vascularization*. Without an axial blood supply to the scaffold, nutrient transport occurs within a maximal proximity of 100–200 μm in highly metabolic tissues (Das and Botchwey, 2011). This concept of axial vascularization has been expanded through the use of vascularized flaps of regenerative tissue with scaffold-guided tissue engineering principles. As such, a tailored approach to flap-based axial vascularization may assist with bridging the gap toward a successful translation of scaffold biomaterials into clinical practice with the potential for a wide range of applications. This process is termed *regenerative matching* axial vascularization. This proposed technique provides a vascularized progenitor to facilitate tissue regeneration in the scaffold (Reichert et al., 2012). The principles of scaffold vascularization are underpinned by our understanding of how normal tissue receives its blood supply, and how this can be manipulated using techniques used in contemporary reconstructive surgery.

A *graft* is a discrete piece of tissue harvested and transferred without an intact arteriovenous network connection to the host and often has a random pattern vascular network. The behavior of a graft following transfer is underpinned by a reliance on the process of angiogenesis, where the existing micro-vasculature of the graft attempts to integrate with the host micro-vasculature surrounding and within the defect. In contrast, a *flap* is harvested and transferred with its arteriovenous network kept in continuity and in modern reconstructive surgery, is commonly performed using an axial pattern approach with a defined arteriovenous pedicle for medium to large volume tissue harvest. Flaps can be defined in terms of the way they are transferred, which is inextricably related to its pattern of blood supply. Local flaps are harvested from a donor site that is immediately adjacent to the defect and have a geometric design with a random pattern of vascularization. Regional flaps may be raised from a donor site that is further from the defect because they are raised on an axial arteriovenous pedicle. They may be transferred free, whereby the vascular pedicle of the flap is detached and anastomosed to target vessels in the region of the defect.

**Figure 1:**
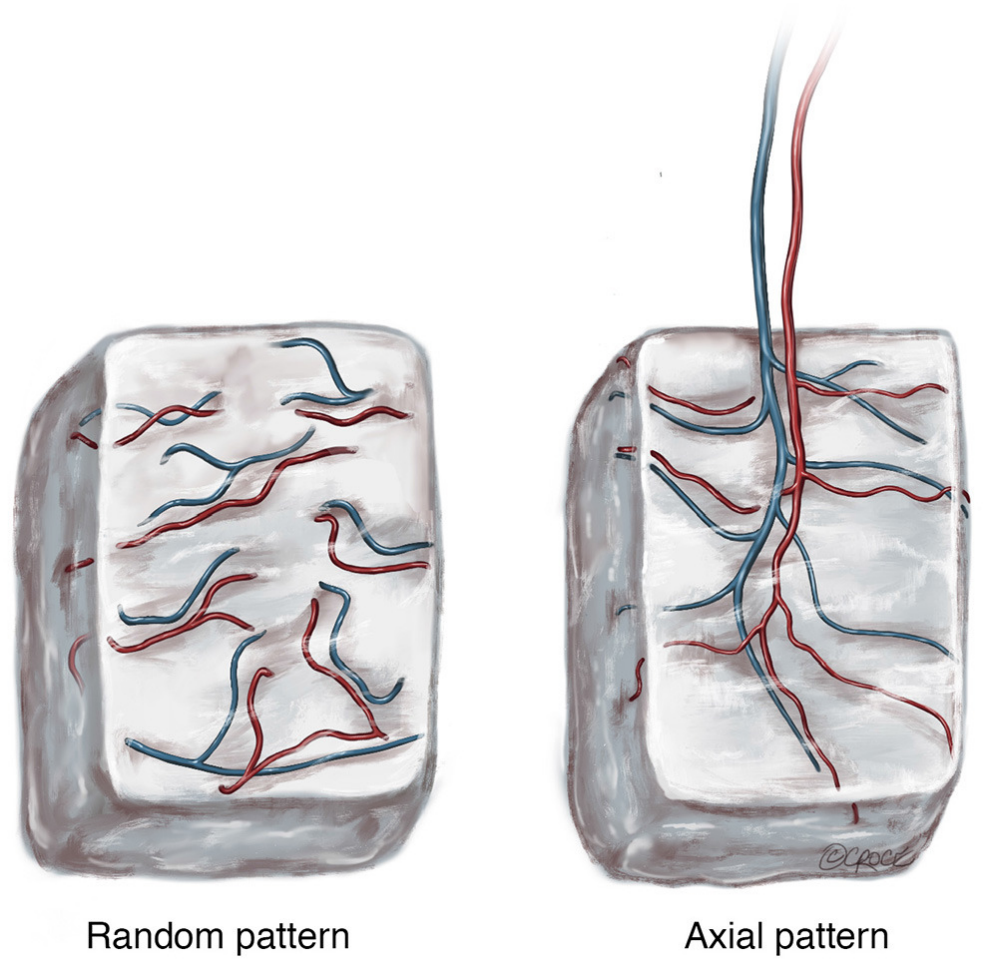
The two patterns of blood supply to tissue following harvest with (Random) or without (Axial) preservation of the arteriovenous pedicle. © Beth Croce, Bioperspective.com.

McGregor and Morgan identified the difference between random pattern flaps and axial pattern flaps in 1973 (McGregor and Morgan, 1973) during the “anatomical revolution” of reconstructive surgery in the 1970s (Taylor and Palmer, 1987). In essence, a *random pattern flap* of tissue is one that lacks any significant bias in its vascular pattern (Figure 1). Alternatively, an *axial pattern flap* is one that has a recognized arteriovenous system running along its long axis (Figure 1). These concepts can be aptly applied to the pattern of vascularization used to induce neo-vascularization of scaffold biomaterials. A random pattern approach to scaffold vascularization is one that does not have a clearly defined arteriovenous axis. On the other hand, an axial pattern approach has a clearly defined arteriovenous axis.

The difference is important because scaffolds with a random pattern of vascularization, such as those implanted in tissue without a clear arteriovenous axis for transfer or those with vascular networks created *ex vivo* utilizing pre-vascularization techniques, function like a “graft” when transferred (or transplanted) into a host defect. The behavior of a graft following transfer is underpinned by a complete reliance on the process of angiogenesis within the host recipient site, where the existing micro-vasculature of the graft attempts to integrate with the host micro-vasculature surrounding and within the defect. Consequently, scaffolds with a random pattern of vascularization are subject to unpredictable angiogenesis and often, living tissue within the scaffold (impregnated stem cells etc.) will become ischaemic following transfer, subsequently undergoing necrosis before sufficient angiogenesis occurs. This is exemplified in a study by Schliephake et al. (1995), where a porous hydroxyapatite (HA) matrix was implanted in the sub-periosteal plane of the buccal aspect of the ascending ramus of the mandible and transferred as an avascular HA-bone composite graft to the ipsilateral horizontal mandibular ramus. Although there were signs of bone ingrowth from the host into some of the transplanted specimens, this was variable and soft tissue invasion and associated resorption was also seen, particularly in the graft furthest from the host bone. As is apparent clinically, only small volumes of tissue can be successfully transferred using this technique and this would be regardless of whether growth factors (BMP-7) or other biological additives (mesenchymal stem cells etc.) are incorporated in the construct. The key advantage of the axial vascularization approach is that the micro-vasculature grows into the tissue along a defined axis that can be surgically transplanted to a different anatomical site without interrupting the blood supply. This preserves tissue viability and optimizes the regenerative potential of tissue that is transplanted.

The scientific principles underpinning the *in vivo* bioreactor concept have been known for some time. Research by Hutmacher et al. dates back to 1994 with the use of a HA scaffold implanted into the iliac crest of Göttinger minipigs (Schliephake et al., 1994). Much experimentation using the *in vivo* bioreactor approach has occurred for bone regeneration over the last few decades in a range of animal models. Expanding on the *in vivo* bioreactor concept, Holt et al. in 2005 introduced a pre-clinical model of “axiality” with the use of a vascular pedicle to neo-vascularize a coralline scaffold *in vivo* (Holt et al., 2005). The principle of this approach is based on the idea of the innate self-regenerative ability of the host. Following a period of *in vivo* incubation within the host, the scaffold becomes vascularized by the pedicle and is then transferred to the defect, which may be remote from the incubation site. The means by which tissue regeneration occurs is not clear in this case but is related to the introduction of vascularity and all that this brings. This approach is used in reconstructive surgery and is known as *prefabrication* (Pribaz and Fine, 1994).

**Figure 2:**
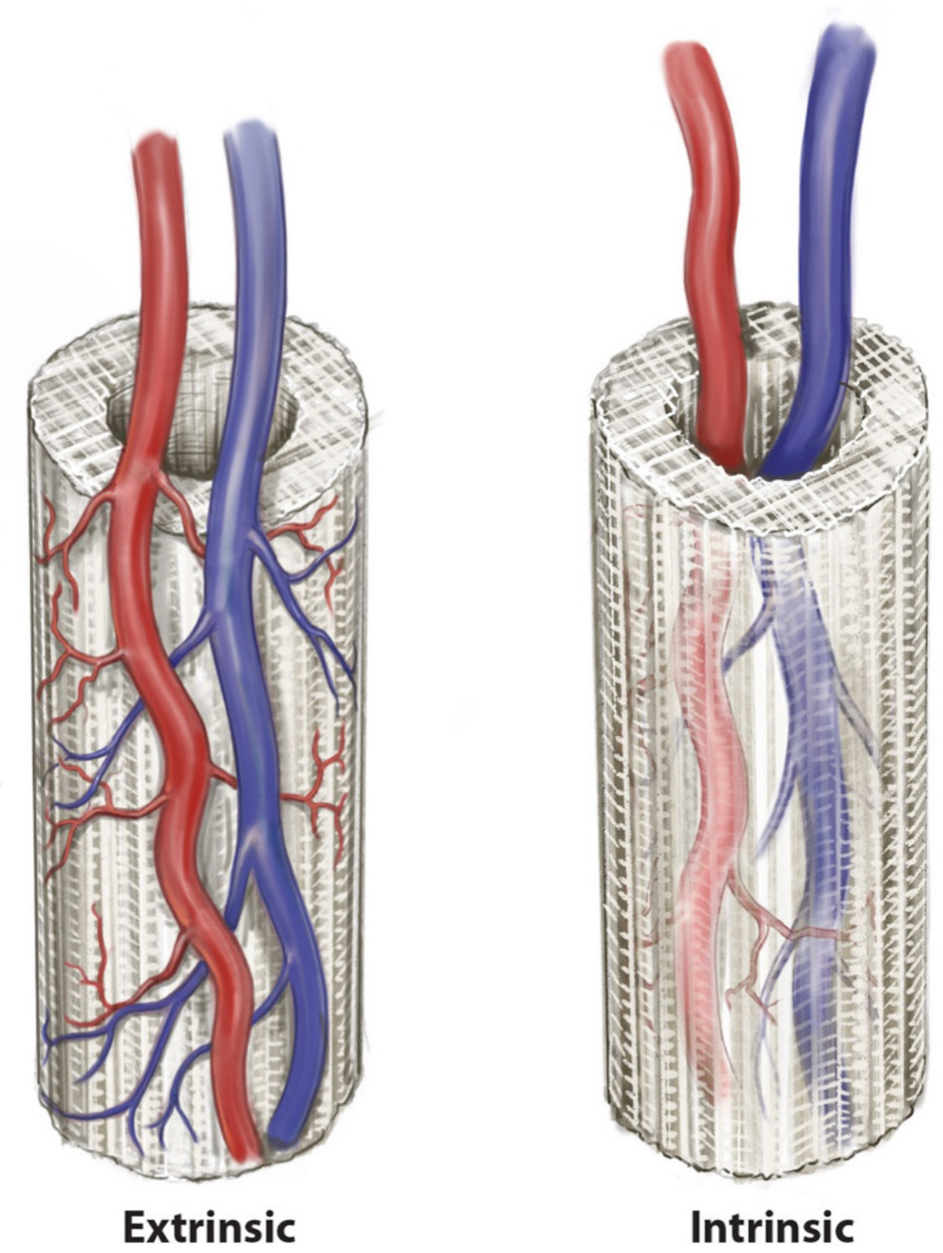
Two patterns of axial vascularization for scaffold constructs, the *intrinsic* and *extrinsic* approaches. © Beth Croce, Bioperspective.com.

The location of the arteriovenous axis (or its integrated tissue network) in relation to the scaffold is clearly important to the success of neo-vascularization. As discussed, scaffold neo-vascularization is predicated on sufficient and uniform neo-vascularization, with cell function severely limited by a diffusion distance >200 μm from a blood vessel. If a scaffold is implanted into a defect and allowed to neo-vascularize from a bed with a random vascular pattern, deficiencies in tissue integrity proportional to the distance from the neo-vasculature may be expected (Horch et al., 2012). Therefore, the introduction of axiality to the vascular network of a scaffold provides a more robust ability to generate new vascular networks and promote mature bone tissue regeneration. This can be performed in an *extrinsic* (external) or *intrinsic* (internal) fashion as defined by the relationship between the scaffold and the pedicle (Kneser et al., 2006b). Extrinsic and intrinsic vascularization approaches (Figure 2) have both been used in the pre-clinical and clinical studies (Warnke et al., 2004; Kokemueller et al., 2010; Horch et al., 2014; Wiltfang et al., 2016). However, for relatively large scaffolds whose purpose is to produce a large uniform volume of regenerate bone, extrinsic vascularization shows only limited success. This is because the potential for angiogenesis, and therefore tissue regeneration, in the center of the scaffold is limited as this is the part of the construct that is furthest from the axial blood supply (Horch et al., 2012). Intrinsic vascularization can overcome this limitation by introducing an axial blood supply to the center of the scaffold with more uniform angiogenesis and tissue regeneration (Leibig et al., 2016). The approach encompasses the use of an arteriovenous conduit through the scaffold and depending on placement of the scaffold, also combines with an extrinsic pattern of vascularization by default, by virtue of coverage by soft tissues at the periphery of the scaffold, which are known to enhance neo-vascularization (Weigand et al., 2015).

## Vessel-Based Axial Vascularization (VBAV)

As discussed above, a discrete arteriovenous pedicle can be used to neo-vascularize a scaffold. Typically, this would be based on the intrinsic approach to axial vascularization, with or without a random-pattern extrinsic neo-vascularization from the surrounding soft tissue. Arteriovenous loops have been used extensively to vascularize tissue engineered constructs for a range of tissue types including adipose tissue (Matsuda et al., 2013), cardiac muscle (Tee et al., 2012), skeletal muscle (Messina et al., 2005; Bach et al., 2006), fibrous tissue (Mian et al., 2000; Beier et al., 2009) and bone (Kneser et al., 2006a; Beier et al., 2011; Rath et al., 2011, 2012; Eweida et al., 2014). The idea was originally described by Erol and Sira (1980) and later expanded upon by Tanaka et al. (2000). An arteriovenous loop is created by the microvascular anastomosis of the artery from a vascular pedicle to its venae comitantes via an interpositional vein graft. In engineering bone, such an arteriovenous loop would be applied to the internal surface of a scaffold, inducing host cell infiltration and neovascularization with subsequent replacement of the scaffold by autologous regenerate bone (Leibig et al., 2016). Much work over the last decade has been undertaken by Kneser et al. to validate this approach experimentally for bone regeneration (Arkudas et al., 2007, 2010, 2012; Polykandriotis et al., 2007; Beier et al., 2010; Rath et al., 2012) and successful clinical translation has been undertaken as well by this group (Horch et al., 2014).

**Figure 3:**
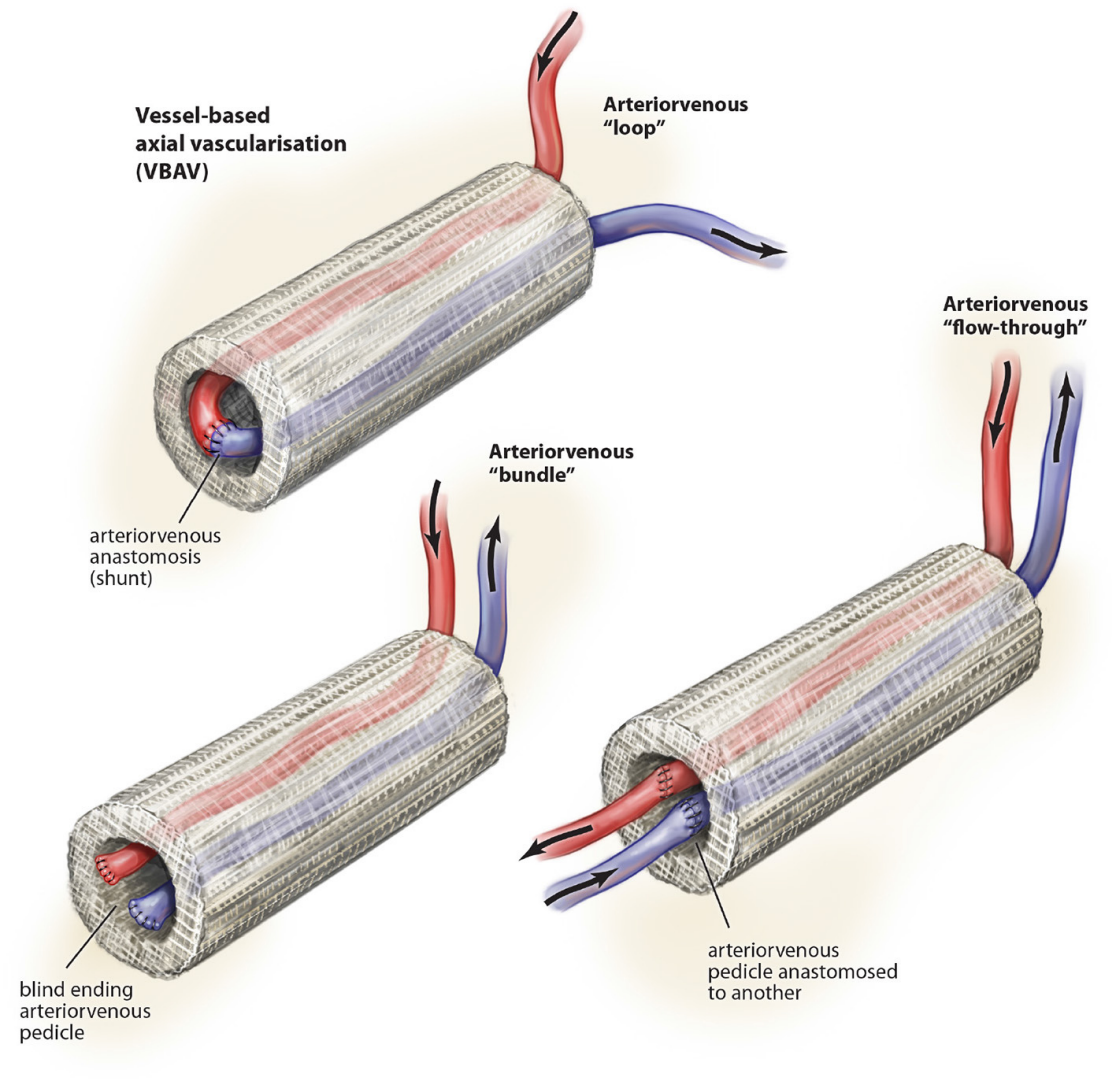
Described variations of vessel-based (VBAV) techniques for axial vascularization of scaffold constructs. © Beth Croce, Bioperspective.com.

There are a variety of different vessel combinations (Figure 3) have been used in of which the arteriovenous loop is the most common (Leibig et al., 2016) and has been used in a range of different scaffold and hydrogel tissue regeneration including fibrin, Matrigel, PLGA, and Matriderm (Leibig et al., 2016). Key alternatives include the arteriovenous bundle (Tanaka et al., 2003; Muller et al., 2011; Dong et al., 2012; Yang et al., 2013) and the arteriovenous flow-through (Gill et al., 1998; Tanaka et al., 2003) configurations.

Most studies suggest superiority of the arteriovenous loop over the bundle and flow-through configurations (Tanaka et al., 2003; Dong et al., 2012), which may relate to the shear stress across the interpositional vein graft (Polykandriotis et al., 2007).

There is emerging clinical experience with VBAV techniques to generate bone (Kokemueller et al., 2010; Horch et al., 2014) and some suggest it may be time to start a clinical trial (Eweida et al., 2015). Horch et al. (2014) were the first to clinically translate the arteriovenous loop with the reconstruction of traumatic tibial defect and a distal radius osteomyelitic defect. In the distal radius defect, they used the arteriovenous loop to axially vascularize a ßTCP/HA scaffold intrinsically with impregnated autologous bone marrow aspirate from the iliac crest and fibrin glue. This latter patient had an excellent clinical recovery and radiographic union of the defect was achieved at 14 months follow up. Another study by Kokemueller et al. (2010) utilized an arteriovenous bundle to axially vascularize four tubular ßTCP scaffolds through the thoracodorsal arteriovenous bundle. The scaffolds were buried in the lattisimus dorsi muscle during prefabrication for 6 months before transfer with the thoracodorsal arteriovenous bundle in a titanium cage to reconstruct an osteomyelitic defect of the mandible. A year following surgery there were no signs of infection or rejection of the implant and a satisfactory clinical outcome was achieved.

There are several advantages to vessel-based approaches to axial vascularization. The potential donor sites are multiple throughout the human body, and by using only the arteriovenous pedicle the donor-site morbidity is minimal. Although the two-stage approach with a delay period for prefabrication is typically used, for small defects it may not be necessary (particularly if there is sufficient stability across the bone with a bridging plate or otherwise). Another key advantage to VBAV is the reproducibility and surgeon familiarity associated with using an arteriovenous circuit. Although the angiogenic potential of the arteriovenous loop is well-established, the approach requires the osseoinductive scaffold to direct the growth of regenerate bone. This process can be assisted with impregnation of the biomaterial with different growth factors and mesenchymal stem cells, although the latter requires an additional phase of delay to grow these cells in culture *ex vivo*. For further enhanced generation of bone, it may be that a vascularized flap of regenerative tissue, such as a corticoperiosteal flap to regenerate bone, may be better suited in the clinical setting and specifically, where a large volume of bone is required.

## Flap-Based Axial Vascularization (FBAV)

**Figure 4:**
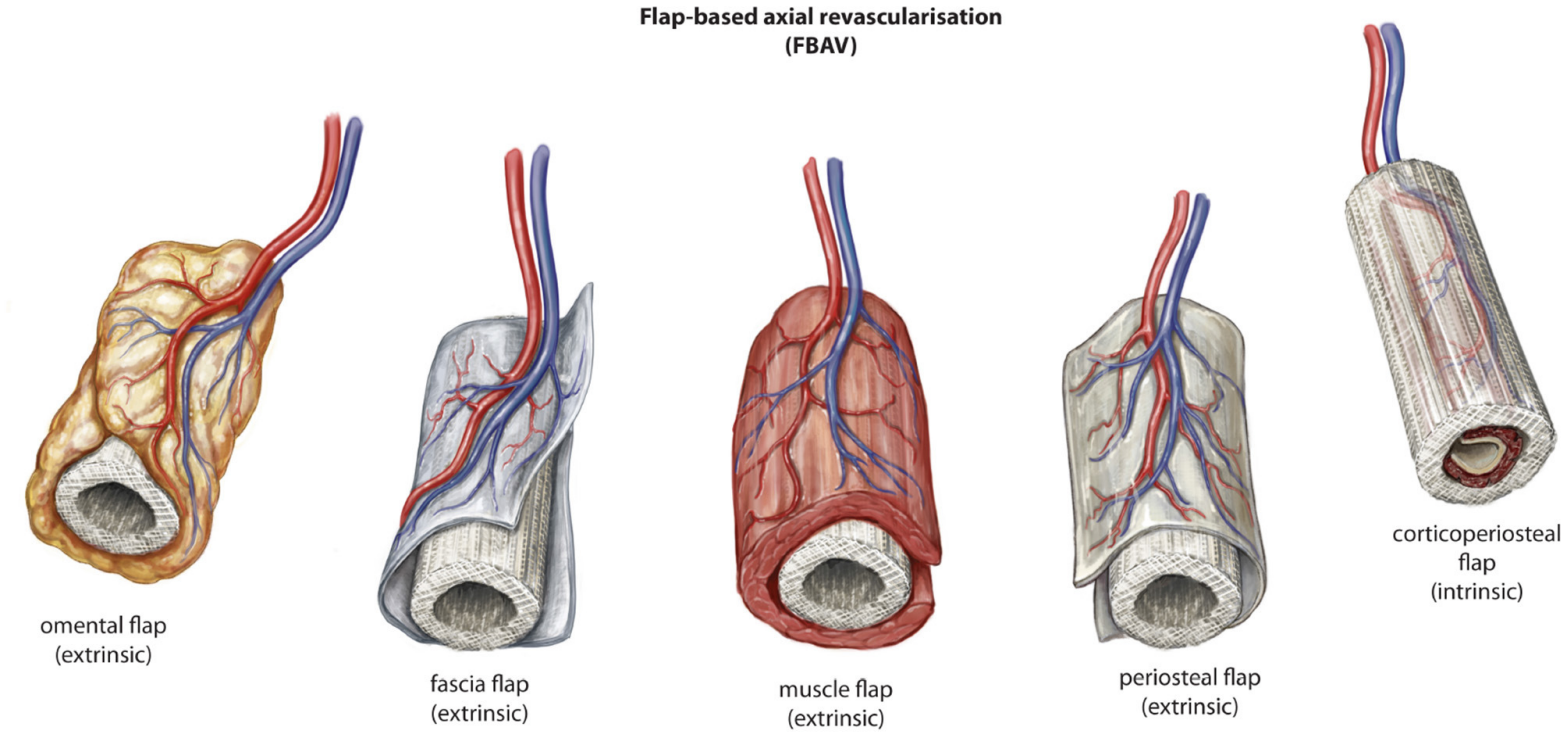
Described variations of flap-based (FBAV) techniques for axial vascularization of scaffold constructs for bone regeneration. © Beth Croce, Bioperspective.com.

The use of flap-based options for axial vascularization is underpinned by the principle that a flap of tissue with an established vascular network, also defined as a vascular axis, progenitor cells with regenerative potential and growth factors is an efficient means for tissue regeneration by extrinsic or intrinsic scaffold vascularization. The approach can facilitate a single-stage or two-stage process for generation of new vascularized bone prior to detachment and transfer to a defect in a remote anatomical site. A variety of different tissue types have been exploited in this regard for scaffold vascularization in the setting of large volume bone regeneration (Figure 4).

### Omental Flaps

The angiogenic properties of omentum have been identified for centuries (Zhang et al., 1997). This has led to its experimental use as an approach to axial vascularization of scaffold biomaterials for regeneration of bone (Jacinto-Tinajero et al., 2014; Wiltfang et al., 2016) and other tissues (Li et al., 1997; Baumert et al., 2007). In a pre-clinical canine study, Jacinto-Tinajero et al. (2014) evaluated the omental flap as an approach to extrinsic vascularization of collagen type-1 sponges seeded with a combination of demineralized bone powder, calcium chloride, thrombin and platelet rich plasma in an effort to generate heterotopic bone. In all animals they were able to demonstrate production of viable trabecular bone within 4 months without growth factor stimulation. In a second arm of the study where transforming growth factor beta-1 (TGF-ß1) was used, bone growth was observed in 80% of scaffolds within one month. The success of the omental flap in this study probably relates to its angiogenic potential (Zhang et al., 1997), although a role for the mesenchymal stem cells resident in omental tissue is likely (Zuk et al., 2001). There is some evidence that the intense angiogenic nature of the omentum may relate to the significantly higher expression and production of VEGF by omental tissue when compared with other organs (Zhang et al., 1997). Reviewing pre-clinical studies related to omental flaps in bone tissue regeneration, alongside an osseoconductive scaffold, there is a clear role for native mesenchymal stem cells growing into the scaffold in synergy with new blood vessel formation guided by the omentum.

Clinical experimentation with an omental flap for extrinsic scaffold vascularization was described by Wiltfang et al. (2016). Using a contoured titanium mesh cage loaded with bone mineral blocks, iliac crest bone marrow aspirate and BMP-2, they performed a two stage approach using the gastrocolic omental flap based on the right gastroepiploic artery and vein. Three months after implantation in the omentum, the construct was harvested on its vascular pedicle and transferred to a central defect in the mandible. Viable bone activity was observed on bone scintigraphy during the postoperative period and viable osteocytes were observed histologically following open biopsy after a minor mucosal dehiscence over the titanium scaffold. It is expected that the extrinsic axial vascularization approach is the most suited to the omentum and that a period of incubation would be required prior to harvest and transplantation. Future research should evaluate the role of omentum in scaffold vascularization when compared with alternative strategies such as the arteriovenous loop and other tissue types.

### Fascial Flaps

Fascia has a dense vascular network and is commonly harvested with other tissue types to perverse their vascularity (Cormack and Lamberty, 1984). In scaffold pre-lamination, fascia has been used with some success pre-clinically for bone regeneration (Fan et al., 2014). In a study by Fan et al. (2014) a fascial flap was used in the tibia of rhesus monkeys to extrinsically vascularize a ßTCP scaffold that also had an intrinsic vascularization approach with an arteriovenous bundle. The construct was created in a single stage. The scaffold was seeded with autologous mesenchymal stem cells cultured from the animal and a control group of scaffold without vascularization was also used in the study. A combination of extrinsic/intrinsic vascularization approach with scaffold and MSCs generated the best outcomes as measured by the histological quality of regenerate bone and radiological evidence of bone healing. Also, vascularity was more pronounced in areas of bone regeneration. A second group with extrinsic fascial vascularization of the scaffold seeded with MSCs also performed well, despite the absence of an intrinsic axial vascularization approach. This highlights the importance of vascularity in this setting yet also that there may be no significant benefit between an intrinsic or extrinsic pattern of axial vascularization for defect of this size and volume comparatively.

In a clinical setting, fascial flaps can be found in a range of anatomical locations, all with minimal donor site morbidity. They could easily conform to the shape of a scaffold construct and have been used for either or both intrinsic (Giessler et al., 2007) and extrinsic (Leonhardt et al., 2009; Fan et al., 2014) vascularization. Further work is required to explore the potential osteogenic characteristics of fascial flaps in the pre-clinical setting prior to clinical translation, although it would appear a combination of fascial flap as an extrinsic approach alongside an intrinsic (arteriovenous loop) vascularization could be useful given the results of the study by Fan et al. (2014).

### Muscle Flaps

Muscle tissue is highly vascular and is a well-known source of mesenchymal stem cells which plays an important role in fracture healing (Chan et al., 2012). From a reconstructive point of view, there is experimental evidence that muscle provides more robust vascularity to a bone defect than fasciocutaneous tissue (Harry et al., 2009) and improved bone healing in case of a fracture yet even more importantly to treat a non-union or large bon volume defect due to tumor removal (Harry et al., 2008). This is presumed to be secondary to the micro-environment of muscle which can sustain osteogenesis through growth factor stimulation (Chen et al., 2004). This feature is key to large bone volume healing and exploited in scaffold prefabrication. Stromal cells that arise from muscle tissue are superior in their osteogenic ability when compared with skin and adipose tissue cells. They have near proliferation and differentiation equivalence to BMCPs in their ability to generate bone (Evans et al., 2009). Experimentation to date with bone-related scaffold biomaterials confirms a role for muscle flaps in axial vascularization. Terheyden et al. have evaluated this concept extensively for mandible reconstruction (Terheyden et al., 1999, 2001a,b). In a porcine model, they implanted an osseoinductive implant (xenogenic bone minerals and BMP-7) into the latissimus dorsi muscle in close proximity to the vascular axis. After 6 weeks, the pre-laminated flap was transferred to reconstruct a mandibular defect and internally fixated. Twelve weeks following transfer the bone was assessed radiographically and histologically at sacrifice with viable bone identified (Terheyden et al., 2001b). Other groups have also experimented with muscle flaps as a means to vascularize scaffolds and confirm the role of this approach in the pre-clinical arena, although some studies suggest muscle is inferior to periosteum for bone regeneration (Huang et al., 2002; Brey et al., 2007).

Clinical translation of muscle flaps for axial vascularization in bone regeneration has been successful to date in two case studies, although significant pro-osteogenic factors were used alongside a titanium cage to help guide bone regeneration. In 2004 Warnke et al. (2004) implanted a titanium mesh scaffold filled with bone mineral blocks, recombinant bone-morphogenic protein-7 (BMP-7) and bovine collagen type-1. Autologous bone marrow aspirate from the iliac crest was used as well as a source of undifferentiated precursor cells for targeted osteogenic differentiation by BMP-7. The scaffold construct was implanted into the latissimus dorsi muscle. Seven weeks later, the pre-laminated scaffold was transferred to reconstruct a sub-total mandibular defect. It is likely the success of this approach derived from a combination of factors, although it seems plausible that sustained bone volume in the construct arose from vascularity—a concept at core to the reconstruction of the mandible with vascularized bone tissue rather than bone graft. In 2009 Mesimäki et al. also performed a two-stage reconstruction of a maxillectomy defect using a muscle flap for pre-lamination of a 3-D printed scaffold (Mesimäki et al., 2009). Like Warnke et al. (2004), they used a titanium cage filled with ßTCP and adipocyte stem cells to guide bone tissue regeneration. The scaffold was pre-laminated in the rectus abdominis muscle and harvested as a free flap for reconstruction of the maxilla with good effect. Importantly, the patient eventually underwent successful osseointegration of dental implants 4 months after the initial operation. Key features make this approach for scaffold pre-lamination attractive. Importantly, muscle flaps are readily available across the body and provide a reliable source of axial vascularization with minimal donor morbidity. These features would also be useful in assisting clinical translation, given the clinician familiarity with these types of flaps. Although both reported clinical experiences appear to have been successful, wholesale translation may be restricted by a number of issues. First, the use of a titanium-cage scaffold may suffer the same problems as any alloplast in the craniofacial skeleton including implant failure, exposure or infection. Furthermore, the technique described by both authors involved multiple stages. By Mesimäki et al. (2009), it is unclear if the additional first stage of the procedure is even warranted given the results achieved with autologous bone marrow aspirate to date in other studies (Warnke et al., 2004; Horch et al., 2014). Despite the aforementioned limitations, this approach to axial vascularization has demonstrated clinical translatability.

### Periosteal Flaps

**Figure 5:**
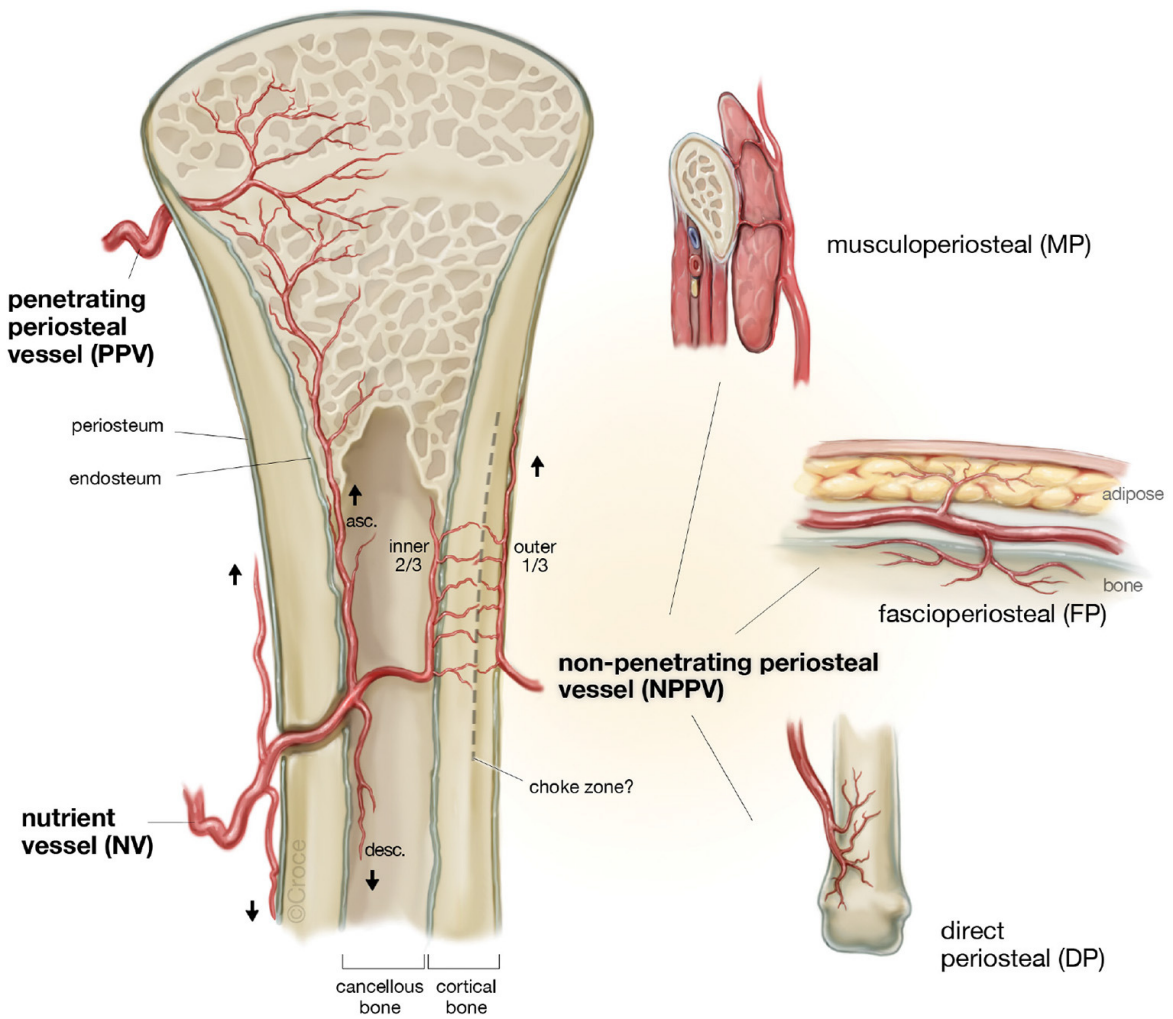
The patterns of blood supply to bone (Reproduced with permission from Sparks et al., 2017).

It is well-known that periosteum carries significant neo-osteogenic capacity and is crucial to fracture healing and the repair of sub-critical sized bone defects (Buck and Dumanian, 2012). It is composed of two well-defined layers, an outer hypocellular fibrous layer and inner cellular layer (cambium layer), where there is a high density of mesenchymal stem cells for new bone generation (Buck and Dumanian, 2012). The current understanding of the blood supply to bone suggests that a direct periosteal (DP) or penetrating periosteal vessel (PPV) blood supply can perfuse the periosteum and adjacent outer third of cortical bone, supporting a robust vascular supply for tissue transfer with numerous potential donor sites (Figure 5) (Sparks et al., 2017). Experimentation to date using periosteal flaps as an axial vascularization strategy has been extensive (Runyan et al., 2010; Li et al., 2012; Han et al., 2014; Ersoy et al., 2015; Nau et al., 2016), yet exclusively performed through an extrinsic approach. A recent study by Nau et al. (2016) evaluated the role of a periosteal flap alone compared with a periosteal flap with a ßTCP scaffold with or without autologous bone marrow derived mononuclear cells (BMCs). This was performed in the setting of a rat femur critical size bone defect with primary fixation. The periosteal flap with scaffold and BMPCs was the most effective in bone regeneration at 4 weeks using radiographic, histological and biomechanical measures of outcome. Interestingly, biomechanical strength was not superior at 8 weeks when compared to the periosteal flap or periosteal flap with scaffold alone. This may indicate an initial advantage in bone regeneration during the early stages of scaffold axial vascularization with adjunctive cellular techniques, but perhaps may be eventually offset by the neo-osteogenic potential of the periosteum at a later time point. Importantly, an additional group with a ligated periosteal flap with scaffold was found to have no new bone regeneration in the scaffold during the study period.

Other experimental studies provide further support for the role of vascularized periosteum as a commanding extrinsic axial vascularization approach for bone regeneration. A study in rabbits by Huang et al. (2002) evaluated the role of different tissue implantation sites (subcutaneous, intra-muscular and sub-periosteal) and the ability to facilitate cartilage and bone regeneration in polycaprolactone (PCL) scaffolds loaded with TGF-ß1 through a fibrin glue carrier. At 6 weeks postoperatively, scaffolds implanted in subcutaneous tissue or muscle had become populated with mesenchymal cells surrounded with abundant fibrovascular tissue. In contrast, the periosteal group was richly populated with chondrocytes and early immature bone formation was observed. Another study by Brey et al. (2007) confirmed these findings with autologous bone graft. Non-vascularized bone graft was implanted either in a periosteal or muscle fascial sleeve and after a 24 week period of pre-lamination, there were no differences in the volume or shape of the tissue formed within the implantation sites. However, those implanted within muscle showed almost exclusive fibrovascular tissue growth whereas the periosteal group showed active new bone formation within the graft. Although robust vascularity is important in bone regeneration, clearly those tissues without specific osteoprogenitor cells (muscle, omentum, subcutis) fail to provide meaningful bone regeneration. The rich source of progenitor cells within the periosteal tissue was therefore the likely source for improved bone regeneration in the periosteal group (Hutmacher and Sittinger, 2003). These cells can adopt an osteogenic phenotype with appropriate signaling by growth factors, an important trigger for which is hypoxia (Hutmacher and Sittinger, 2003). It is likely that vascularity is not sufficient alone to direct bone regeneration and that some type of autologous direction is required.

It would appear that corticoperiosteum rather than periosteum alone may be superior for neo-osteogenesis, as harvesting a thin layer of cortical bone with the periosteum protects the cambium layer from surgically created tissue trauma (Hertel et al., 1994). Much work has been performed to justify this over the last century, most notably with the work of Axhausen in the 1950s where significantly reduced new bone growth in periosteal flaps was identified where periosteum was scraped off rather than with a thin layer of cortical bone (Axhausen, 1956). Therefore, harvest of corticoperiosteum may be important to preserve the neo-osteogenic potential of the periosteum during transfer.

Another concept that is important in interpreting pre-clinical studies is the alteration in periosteal activity between different species (Reichert et al., 2009). Rodents and lower mammals are known to have greater periosteal activity (Brookes and Revell, 1998) than aged large animal models (Reichert et al., 2009). Most studies evaluating axial vascularization studies have focused on small animal models.

## Regenerative Matching Axial Vascularization in Bone Regeneration

Logically, the easiest way to generate tissue should be to introduce to a scaffold a flap of the specific tissue type intended for regeneration along with its progenitor cells. A clear example of this would be to use a *corticoperiosteal flap* to vascularize a scaffold intended for *bone regeneration*. This approach may negate the addition of cell culture based approaches (such as mesenchymal stem cell culture in combination with a scaffold) that aim to enhance bone regeneration. In essence, the osteoegenic potential of the corticoperiosteal flap provides sufficient host-derived osteogenic growth factors, periosteal-derived mesenchymal stem cells and the appropriate extra-cellular environment essential for new bone growth—as seen with fracture healing. This applies the Gilles-Millard principle of a *like for like* reconstruction to surgical prefabrication and is termed *regenerative matching* axial vascularization. Using this approach, it would seem that the highest yield through the *in vivo* bioreactor principle can be achieved whilst incorporating a reliable pattern of intrinsic axial vascularization to the scaffold to sustain the necessary vascularity required for ongoing bone regeneration (Leibig et al., 2016).

**Figure 6:**
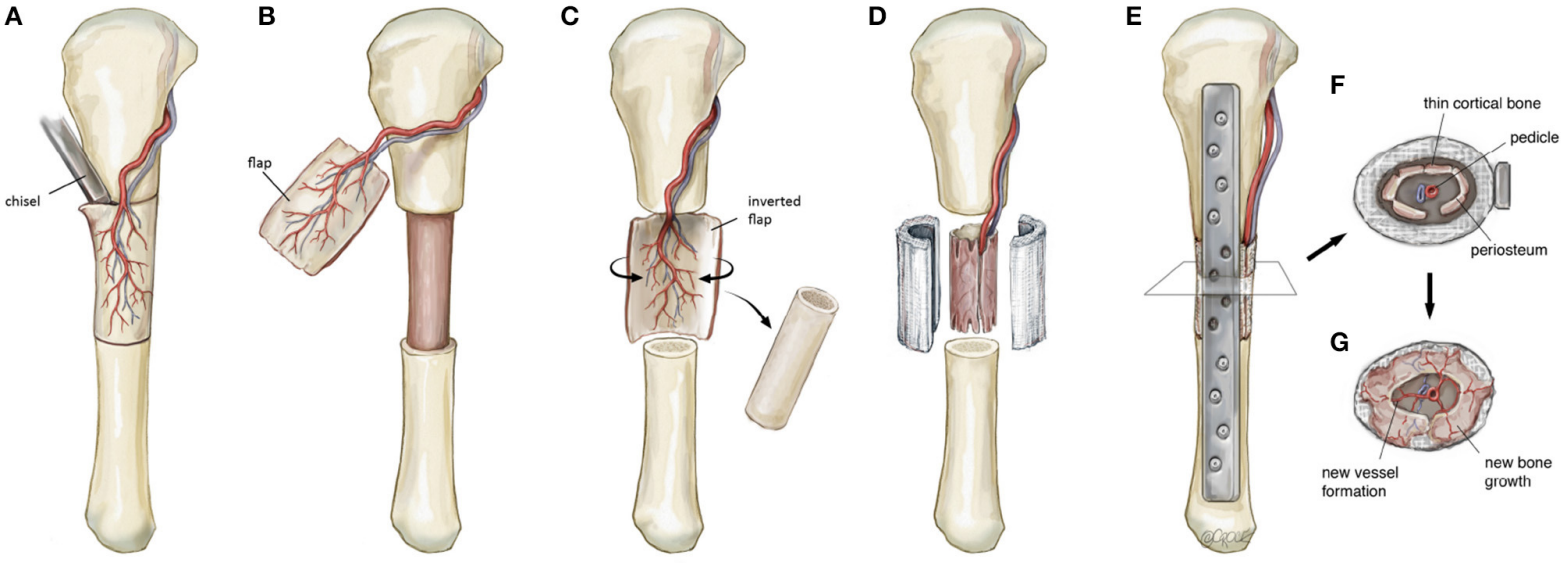
Schematic illustration detailing the steps involved in a regenerative matching approach to scaffold vascularization using a corticoperiosteal flap. Firstly the flap is harvested **(A,B)** prior to placement into the defect and is rolled on itself **(C)** so the corticoperiosteum effaces the scaffold with the periosteum and blood supply internal **(D)**. The bone is then fixated **(E)** and neo-osteogenesis and scaffold vascularization ensues **(F,G)** to generate new autologous bone. © Beth Croce, Bioperspective.com.

The transport of osteoprogentior cells is fundamentally reliant on sufficient and uniform neo-vascularization within the scaffold. It is likely that a pedicled corticoperiosteal flap placed in the internal aspect of the scaffold could exploit much of the same physiological advantages observed in vessel-based strategies (Figure 6). Within the scaffold, a degree of hypoxia external to the location of the flap could potentially drive angiogenesis, as has been shown previously (Yuan et al., 2014). When this process is coupled with neo-osteogenesis and likely driven by periosteal-derived mesenchymal stem cells, both processes may well be synergistic in nature.

There is also a key role for muscle as an extrinsic random-pattern source of vascularision once the scaffold has been implanted in the defect. Nanchalal and co-workers have validated the role of muscle as both a pro-angiogenic (Harry et al., 2009) and pro-osteogenic tissue (Harry et al., 2008). Although the vascularity of muscle is essential for bone regeneration in scaffold biomaterials, it is likely that the pro-osteogenic characteristics of muscle are just as important for bone regeneration observed in experimental (Terheyden et al., 1999, 2001a,b) and clinical studies (Warnke et al., 2004; Mesimäki et al., 2009). Just like in other studies that combine extrinsic and intrinsic approaches to scaffold vascularization (Arkudas et al., 2012; Fan et al., 2014; Weigand et al., 2015), the combination of muscle extrinsically with corticoperiosteum intrinsically may be both optimal and practical for scaffold axial vascularization and bone regeneration.

**Figure 7:**
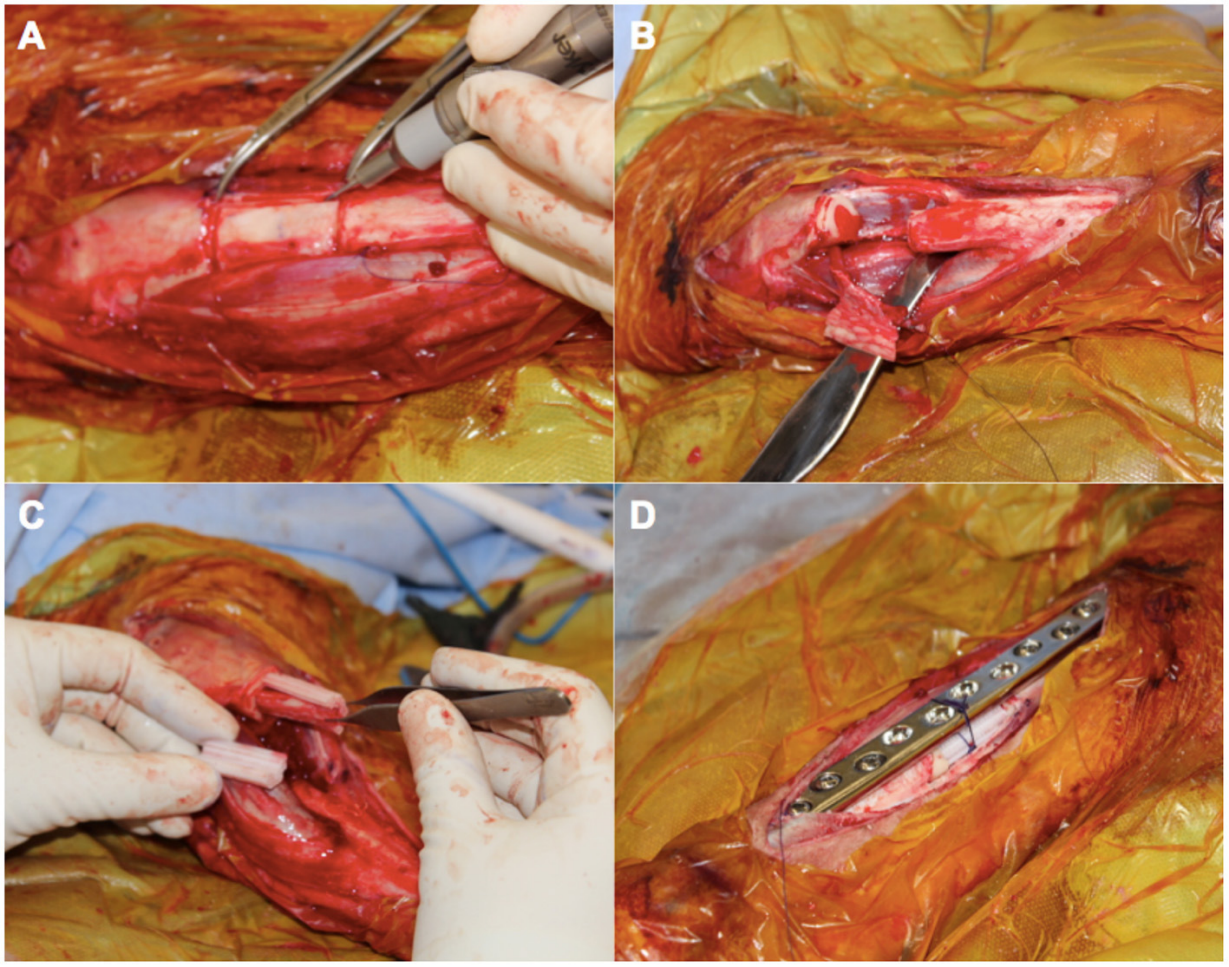
An intra-operative photo series illustrating key components in the surgical approach for scaffold-guided bone regeneration using a corticoperiosteal flap. The flap is marked out sharply and raised off the medial and anterior surface of the tibia using a fine dental burr **(A)** prior to resection of the 3 cm defect **(B)**. The flap is then rolled on itself so the corticoperiosteum effaces the lego-like half cylindrical scaffold with the periosteum and blood supply lying most internal **(C)**. The corticoperiosteal flap with scaffold is then placed within the defect with the residual tibial diaphysis using a dynamic compression plate as internal fixation **(D)**.

To evaluate the feasibility of this novel concept in generating new bone, we used a proof of principle approach incorporating a large animal ovine model (Animal Ethics Approval Number 1600000280) familiar to the research group (Reichert et al., 2011, 2012; Berner et al., 2013, 2015b; Cipitria et al., 2015). Matured sheep (*ovis aries*) closely resemble the clinical setting for a range of reasons including bone architecture and healing as well as osteosynthesis choice (Reichert et al., 2009). Using this model, a corticoperiosteal flap was harvested based on the anterior tibial vascular bundle prior to performing a 3 cm tibial segmentectomy of the mid-diaphysis (Figures 6, 7). This was then placed inside the scaffold and the bone was internally fixated with a dynamic compression plate (Figures 6, 7).

**Figure 8:**
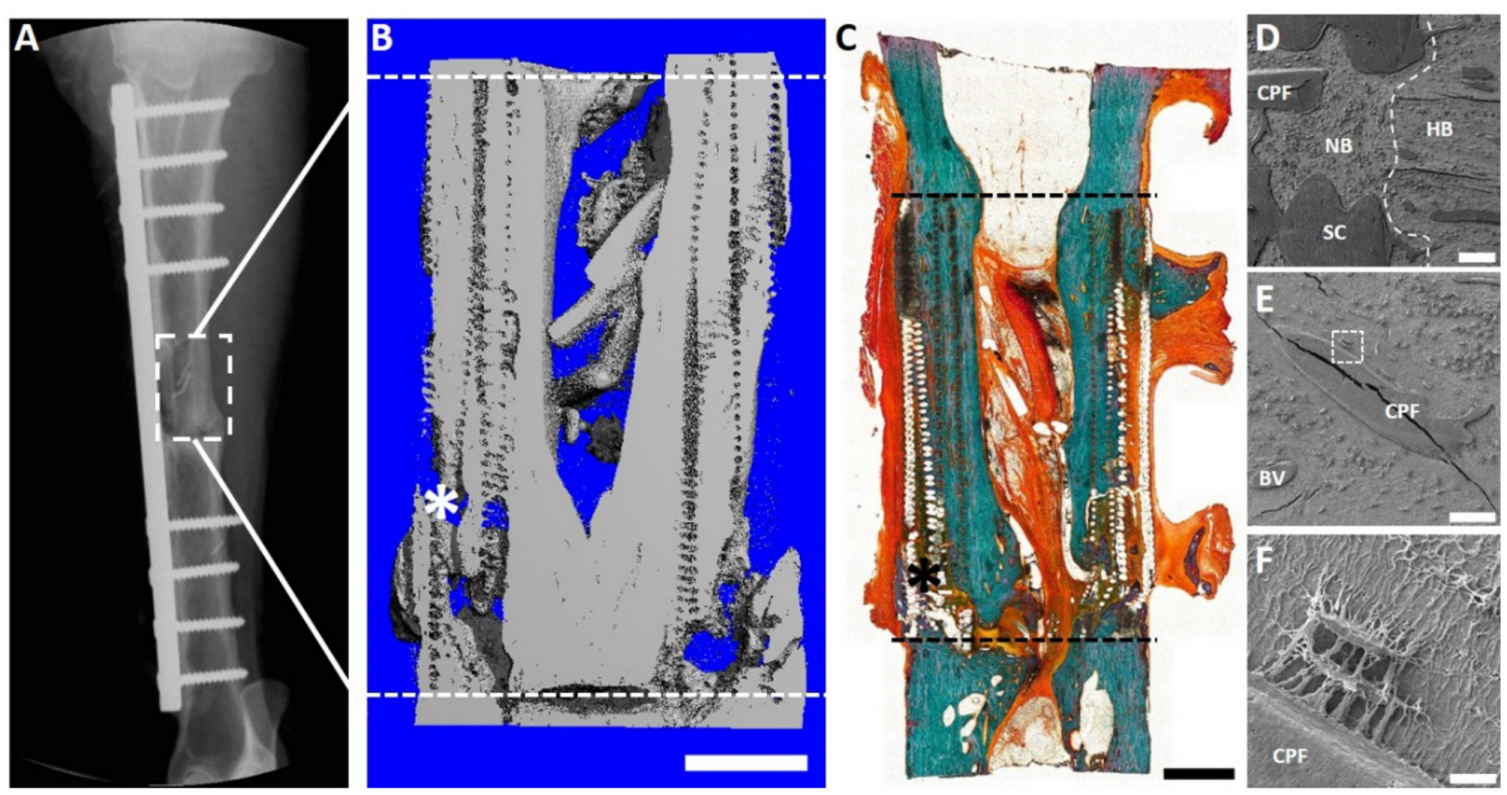
Overview of the 3 cm tibial corticoperiosteal flap results of a pilot study sheep. **(A)** X- ray image at 12 months' time point. **(B)** Sagittal plane of the μCT 3D reconstruction. **(C)** Undecalcified resin section stained with Goldner's trichrome. **(D–F)** Scanning electron microscopic (SEM) images of newly formed bone and interface with host bone. **(D)** Interface of the host bone (HB) with the new formed bone (NB) indicating excellent osteointegration of host bone, scaffold (SC) and the corticoperiosteal flap. **(E)** Higher magnification image showing integration of the corticoperiosteal within the newly formed bone and **(F)** higher magnification of the white square box in image **(E)** showing two osteocytes embedded in the new formed bone and directly attached to the corticoperiosteal flap, indicating direct interaction of corticoperiosteal with osteocytes. Fissures in images **(D,E)** are artifacts resulting from sample preparation. - - - Defect site; ^*^Mechanical testing artifact; Scale bar: **(B)** 5 mm; **(C)** 5 mm; **(D)** 200 μm; **(E)** 100 μm and **(F)** 10 μm.

After 12 months, significant bone healing of the defects were demonstrated using key radiological [Serial x-ray and microcomputed tomography (μCT)], biomechanical (torsional bone stiffness) and histological analyses (Goldner's trichrome and scanning electron micrography) (Figure 8). Importantly, robust bone regeneration is observed throughout the scaffold construct and intramedullary remodeling is nearly complete at the 12 month time point (Figures 8B,C). Scanning electron microscopy (SEM) revealed excellent osteointegration of host bone with the newly formed bone, scaffold and the corticoperiosteal flap (Figures 8D,E). Furthermore, SEM imaging indicated close and direct interaction of the residual corticoperiosteal tissue with osteocytes from the newly formed bone (Figure 8F).

**Figure 9:**
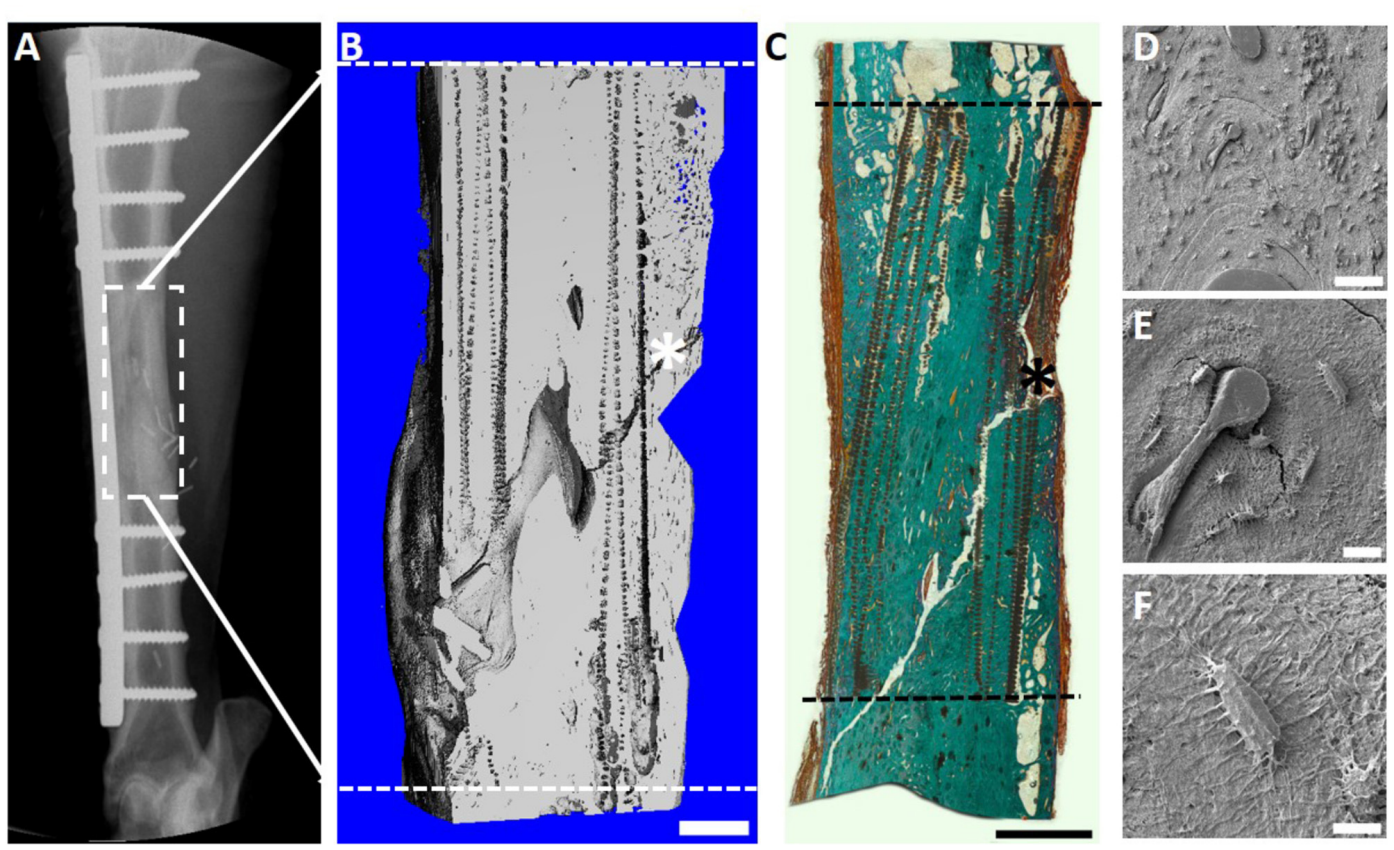
Overview of the 6 cm tibial corticoperiosteal flap results. **(A)** X- ray image at 12 months' time point. **(B)** Sagittal plane of the μCT 3D reconstruction. **(C)** Undecalcified resin section stained with Goldner's trichrome showing excellent osteointegration of host bone, scaffold and newly formed bone. **(D–F)** Scanning electron microscopic (SEM) images showing the osteocyte network of the newly formed tissue. **(D)** Osteointegration of new formed bone in the middle of the defect site. **(E)** Higher magnification image showing secondary osteon formation and osteocytes in the proximity of the osteon's central blood vessel and **(F)** higher magnification of an osteocyte embedded in the newly formed bone matrix. - - - Defect site; Scaffold collapse in **C** resulting from sample processing; ^*^Mechanical testing fissure artifact; Scale bar: **(B)** 5 mm; **(C)** 10 mm; **(D)** 100 μm; **(E)** 50 μm and **(F)** 10 μm.

Given the promising results seen with the 3 cm defect size, a further pilot study was performed using a 6 cm defect size (Animal Ethics Approval Number 1600000280). This was justified as although the *regenerative matching* approach in a 3 cm defect demonstrated satisfactory bone healing, it was recognized the defect size can also be induced to heal using autologous bone graft alone. From a translation point of view, the choice of a 6 cm defect represented a more challenging clinical defect size where an established approach, such as autologous bone graft, is generally not successful and results in a non-union. Complete bone bridging of the 6 cm defect was confirmed with X-ray (Figure 9A), μCT (Figure 9B) and histological evaluation (Figure 9C). The histological results of Goldner's trichrome showed compelling new bone formation and excellent integration of the scaffold and corticoperiosteal flap within the host environment, with complete bridging of the defect site at 12 months (Figure 9C). In accordance with the histological results, SEM images also revealed excellent osteointegration of host bone and newly formed bone. Interestingly and of high clinical relevance secondary osteon formation, as well as osteocytes embedded within the newly formed bone matrix was also observed (Figures 9D–F).

This pilot study provides further support for the potential clinical translation of this approach incorporating the mPCL-TCP scaffold for critical sized segmental bone defects. This approach will be further evaluated in a phase I human clinical trial. This is currently in the process of being set up to evaluate the role of this technique specifically for challenging critical size bone defect reconstruction in the lower limb.

## Concluding Remarks

When incorporating a TEC for bone tissue regeneration, axial vascularization of the construct is key, and central to this process is the synchrony between osteogenesis and vasculogenesis. There are a variety of axial vascularization approaches described for scaffold-guided bone regeneration, including both VBAV and FBAV techniques. However, *regenerative matching* axial vascularization incorporates the advantages of flap-based techniques for neo-vascularization yet also harnesses the *in vivo* bioreactor principle in a more directed “like for like” approach to further assist regeneration of the specific tissue type that is lost, such as a corticoperiosteal flap in critical sized bone defect reconstruction. It is anticipated that extensions of this concept to current scaffold-guided tissue engineering strategies might include further research into the use of cartilaginous flaps for pre-lamination of scaffolds in auricular or joint reconstruction, and synovial flaps for tendon or ligament regeneration.

## Author Contributions

DS was primarily responsible for writing the manuscript and provided assistance in editing for publication. FS was primarily responsible for editing for publication and assisted with manuscript preparation. SS, MS, MW, and DH contributed to manuscript preparation and editing for publication. DS, MW, and DH conceived the idea of the *regenerative matching* axial vascularisation strategy.

### Conflict of Interest

DH is a founder and shareholder in Osteopore (Osteopore International Pty Ltd, Singapore). The remaining authors declare that the research was conducted in the absence of any commercial or financial relationships that could be construed as a potential conflict of interest.
